# Supplementary material for: Incidence of long-term conditions in the Latin American community of London: A validation and retrospective cohort study of 890,922 primary care records, 2005–2022
Source: PLoS One. 2024 Nov 27;19(11):e0312311. doi: 10.1371/journal.pone.0312311 (PMC11602084; doi:10.1371/journal.pone.0312311)
Supplement: S1 File — (DOCX) [file pone.0312311.s001.docx]

# Additional tables and figures supporting the validation of the Latin American cohort.

There were 8,335 Latin American Lambeth residents in the cohort on 27^th^ March 2011 – 3.2% of the total. The dataset-registered population grew to 14,048 at the beginning of 2022 (Figure S2). This compared to 9,352 Latin American-born residents identified in Lambeth in the 2011 census (3.1%) and 9,678 Latin American-identifying residents in the 2021 census (3.1%)^1^. When the Latin American population count was measured at lower super-output area there was a strong correlation between datasets, with the cohort ascertaining 94% of the census-estimated Latin American-born population (Figure S3, regression coefficient 0.94, *R^2^*=0.71). Areas in which there was a relative over-registration of Latin Americans in the cohort tended to be in areas with higher Latin American-born populations. LSOAs with relative under-registration of Latin Americans tended to be close the boundaries of the borough, and with lower Latin American-born populations (Figure S4).

We worked with the Indoamerican Refugee and Migrant Organization to qualitatively assess concordance between self-ascribed Latin American ethnicity and the codes used to identify Latin American patients in the health record.

1 Ethnic group (detailed) - Office for National Statistics. https://www.ons.gov.uk/datasets/TS022/editions/2021/versions/1?showAll=ethnic_group_288a#get-data (accessed 29 Nov 2022).

## Figure S1: Venn diagram showing the types of codes used to identify Latin American people within the dataset


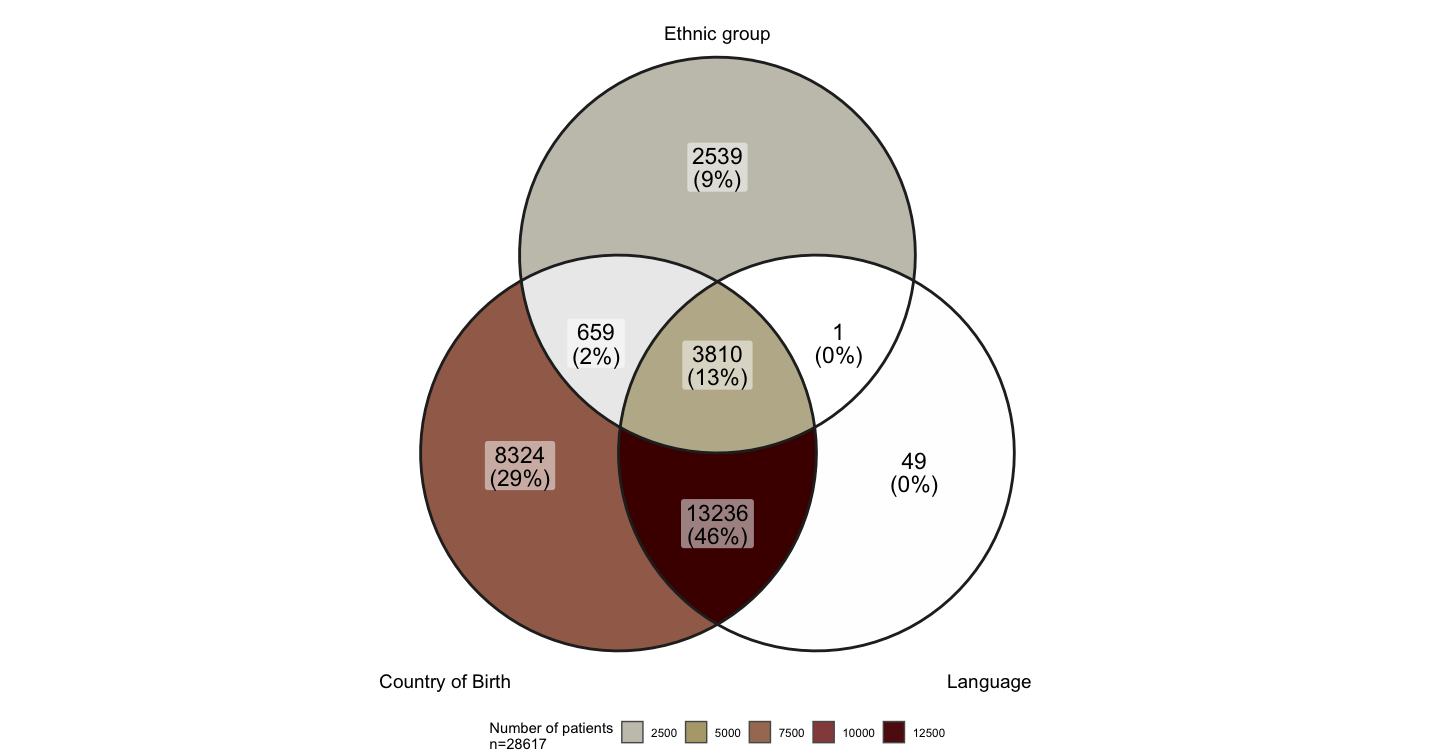


## Figure S2: Number of Latin American patients in Lambeth DataNet, 2005-2022.

*
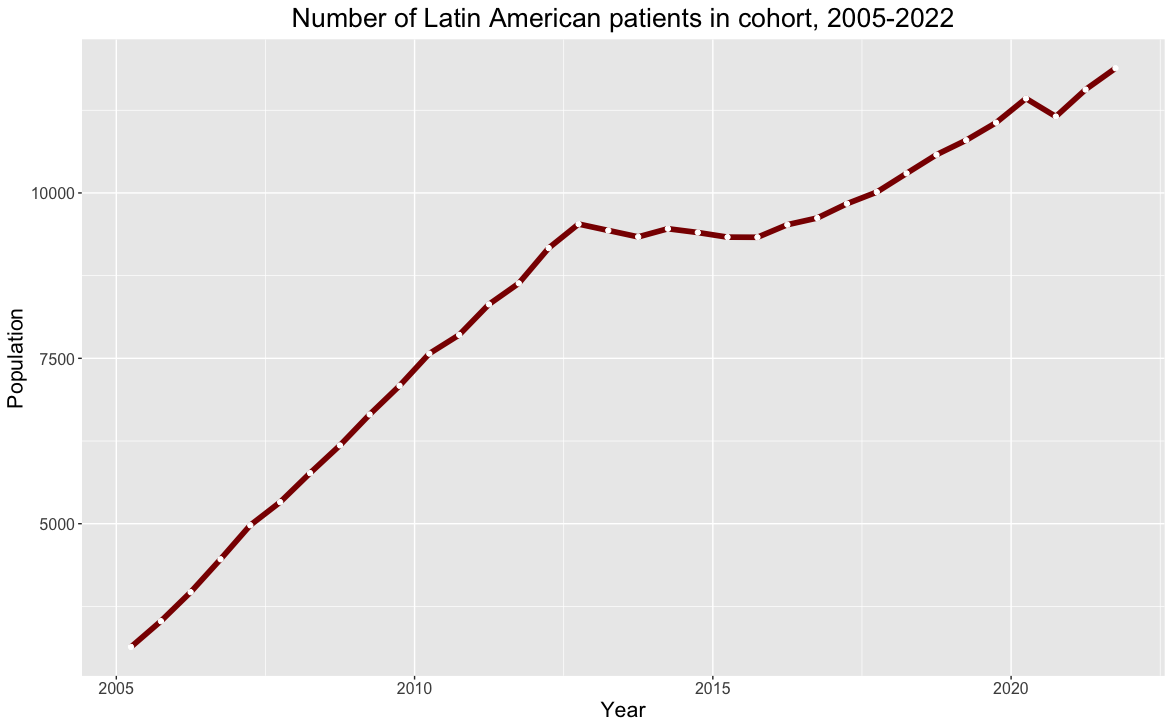
*

## Figure S3: Number of Latin American-born residents of Lambeth on census day 2011. UK Census data, compared to Lambeth DataNet cohort. Each point represents a lower super-output area (LSOA).


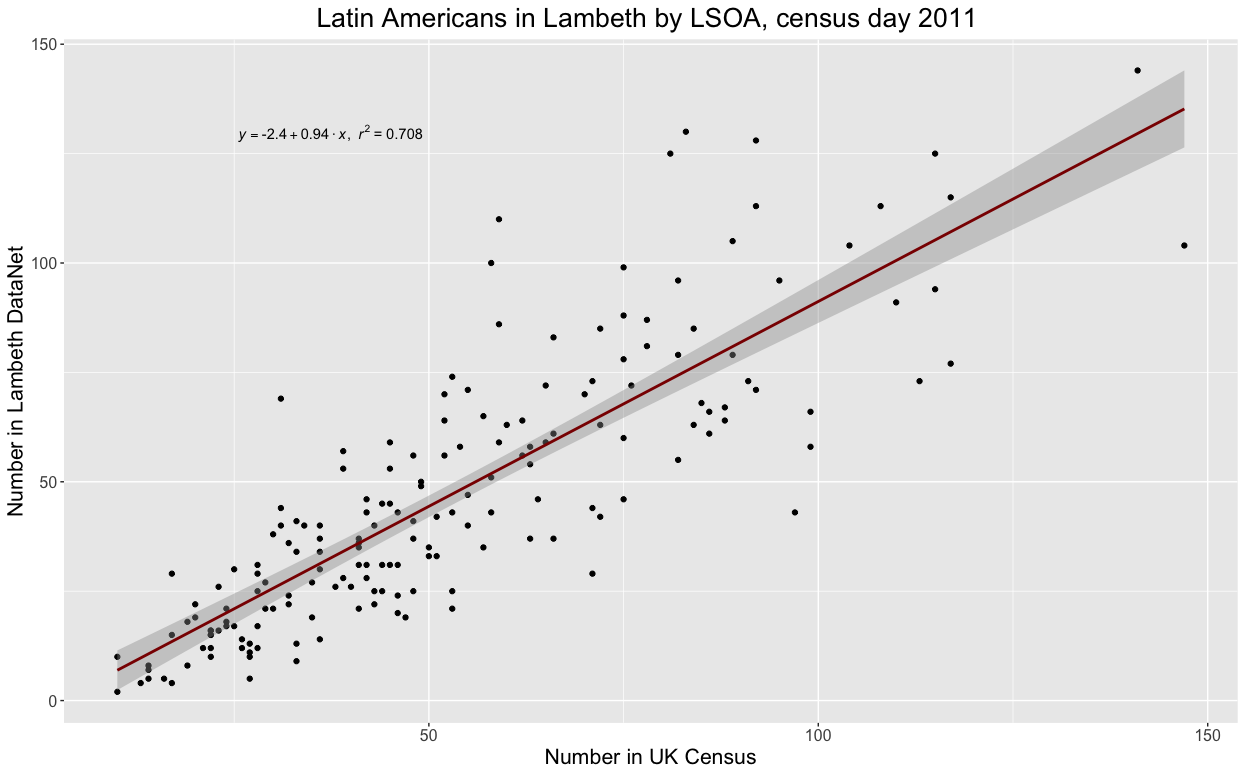


## Figure S4: Choropleth map showing areas of GP over-registration and under-registration of Latin American patients in Lambeth, compared to 2011 census data. Map data is from OpenStreetMap and available under the Open Database License: openstreetmap.org/copyright


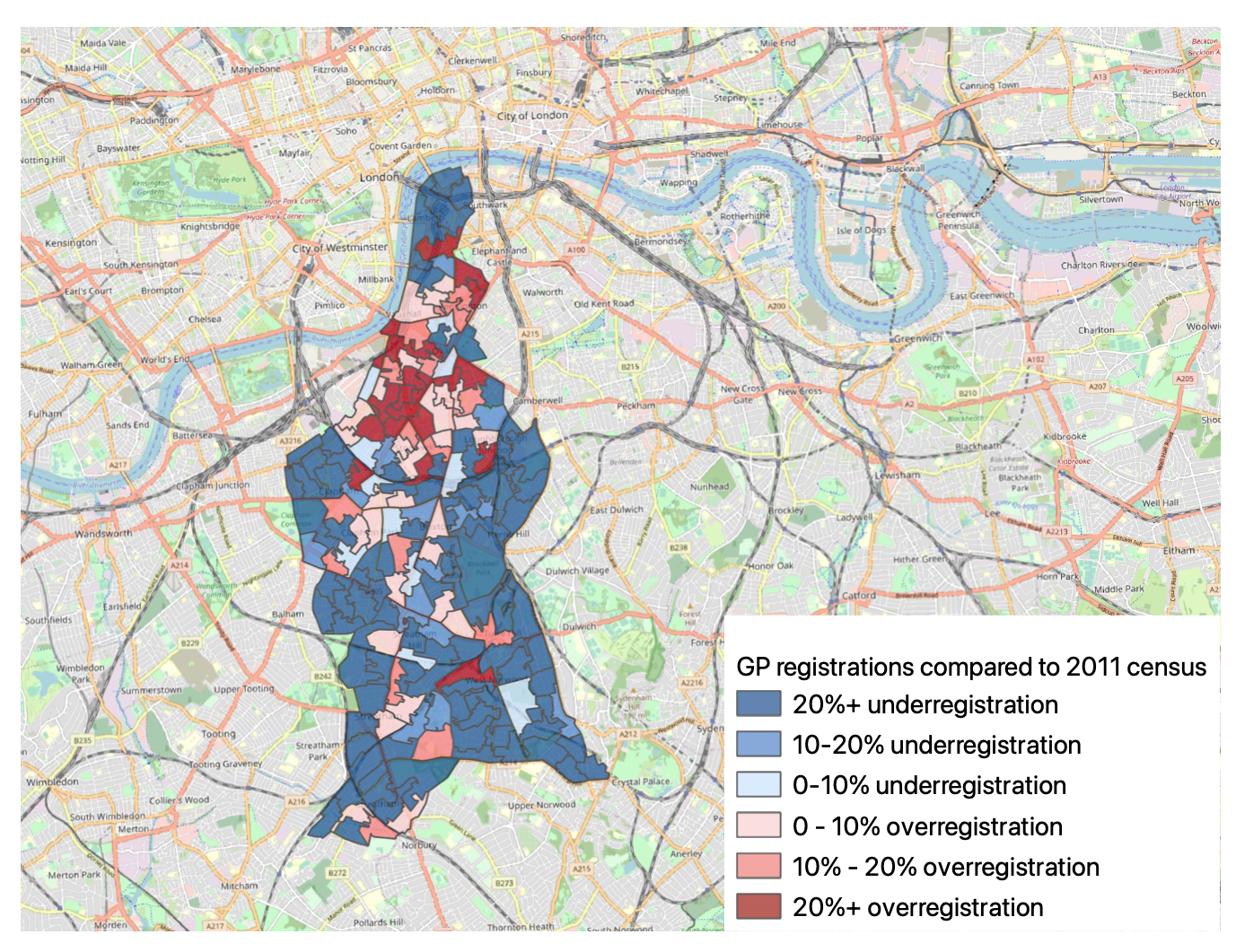


## Table S1: Proportion of Latin American individuals with SNOMED-CT codes for UK Census ethnic groups. Only ethnic groups containing more than 20 people are included.

| **Ethnic group** | | **n (% of total)** | | **Ethnic group** | **n (% of total)** |
| --- | --- | --- | --- | --- | --- |
| White | | 11874 (41.5) | | Other White | 11483 (40.1) |
|  |  |  |  | British | 389 (1.4) |
| Other ethnic group | | 9832 (34.4) | | Any other ethnic group | 9832 (34.4) |
| Mixed/Multiple ethnic group | | 3449 (12.1) | | Other Mixed | 2824 (9.9) |
|  |  |  |  | White and Black Caribbean | 602 (2.1) |
| Black | | 1338 (4.7) | | Caribbean | 997 (3.5) |
|  |  |  | | Other Black | 326 (1.1) |
| Asian/Asian British | | 30 (0.1) | |  |  |
| *Missing* | | *2094 (7.3)* | |  |  |
| **Total** | | **28617 (100%)** | |  |  |
|  |  | |  |  |  |

## Table S2: Proportion of Latin American people in dataset by SNOMED-CT codes for country of birth.

| **Country of birth** | **n (% of total)** |
| --- | --- |
| Brazil | 8524 (29.8) |
| Columbia | 5795 (20.3) |
| Ecuador | 4277 (14.9) |
| Missing | 2589 (9) |
| Bolivia | 1502 (5.2) |
| Dominican Republic | 1055 (3.7) |
| Peru | 889 (3.1) |
| Venezuela | 888 (3.1) |
| Argentina | 758 (2.6) |
| Mexico | 572 (2) |
| Guyana | 560 (2) |
| Chile | 391 (1.4) |
| Honduras | 173 (0.6) |
| Cuba | 150 (0.5) |
| Paraguay | <100 (<0.5) |
| Nicaragua | <100 (<0.5) |
| Uruguay | <100 (<0.5) |
| El Salvador | <100 (<0.5) |
| Guatemala | <100 (<0.5) |
| Costa Rica | <100 (<0.5) |
| Panama | <100 (<0.5) |
| Suriname | <100 (<0.5) |
| **Total** | **28617 (100%)** |
