# Supplementary material for: Incidence of long-term conditions in the Latin American community of London: A validation and retrospective cohort study of 890,922 primary care records, 2005–2022
Source: PLoS One. 2024 Nov 27;19(11):e0312311. doi: 10.1371/journal.pone.0312311 (PMC11602084; doi:10.1371/journal.pone.0312311)
Supplement: S2 File — (DOCX) [file pone.0312311.s002.docx]

## Figure S5: UpSet plot showing the most common thirty long-term conditions (LTCs) and clusters of LTCs recorded in the Latin American and non-Latin American populations, 2005-2022.


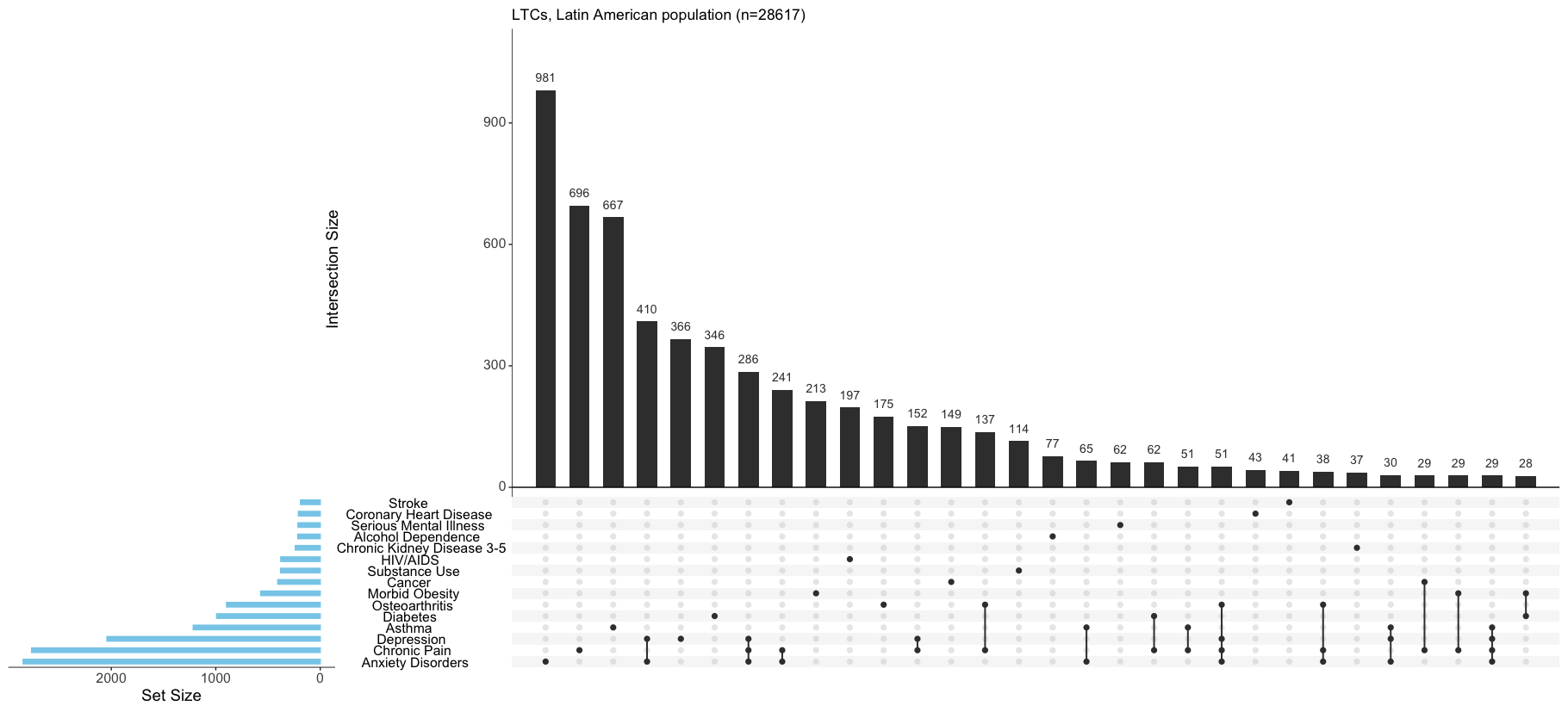


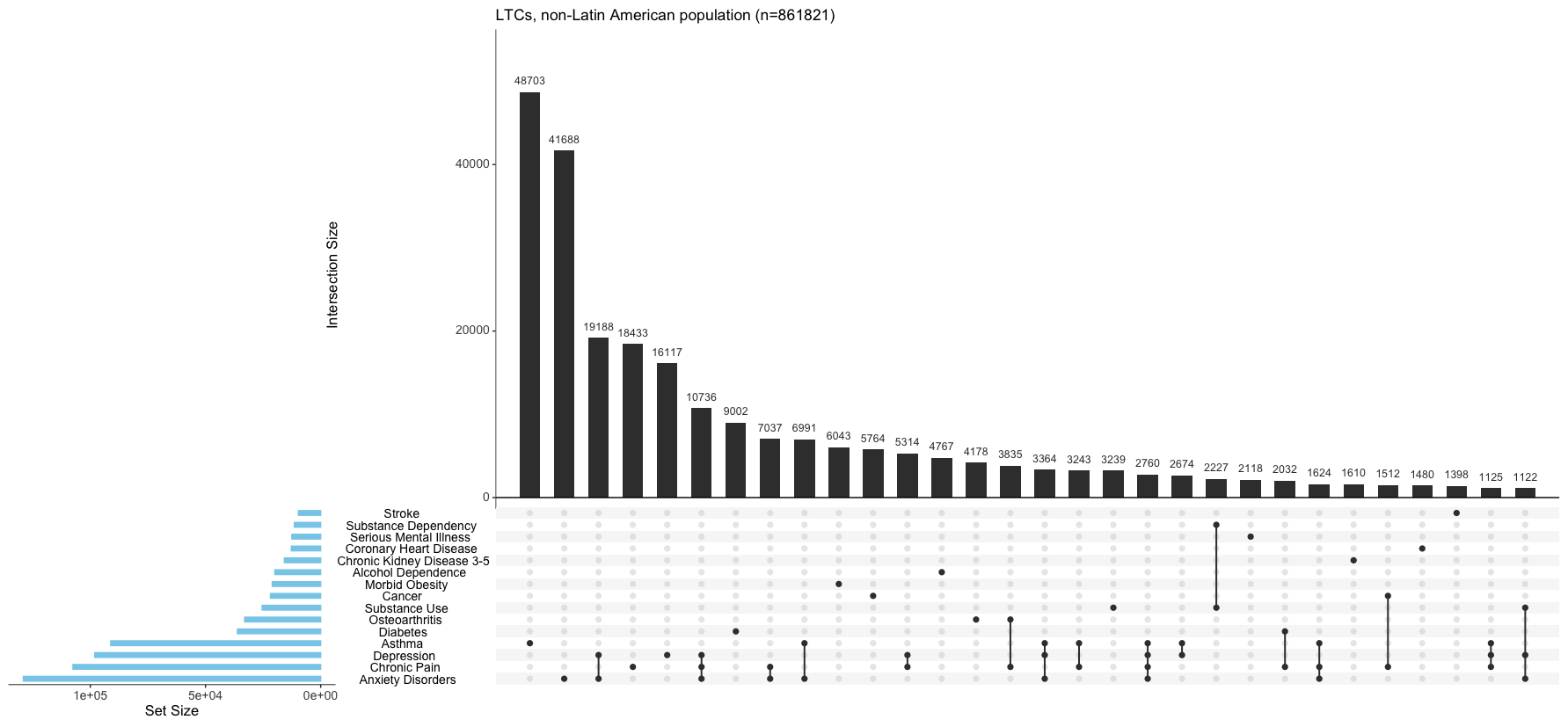


Figure S6: UpSet plot showing the most commonly recorded multiple long term condition clusters in the Latin American and non-Latin American populations, 2005-2022.


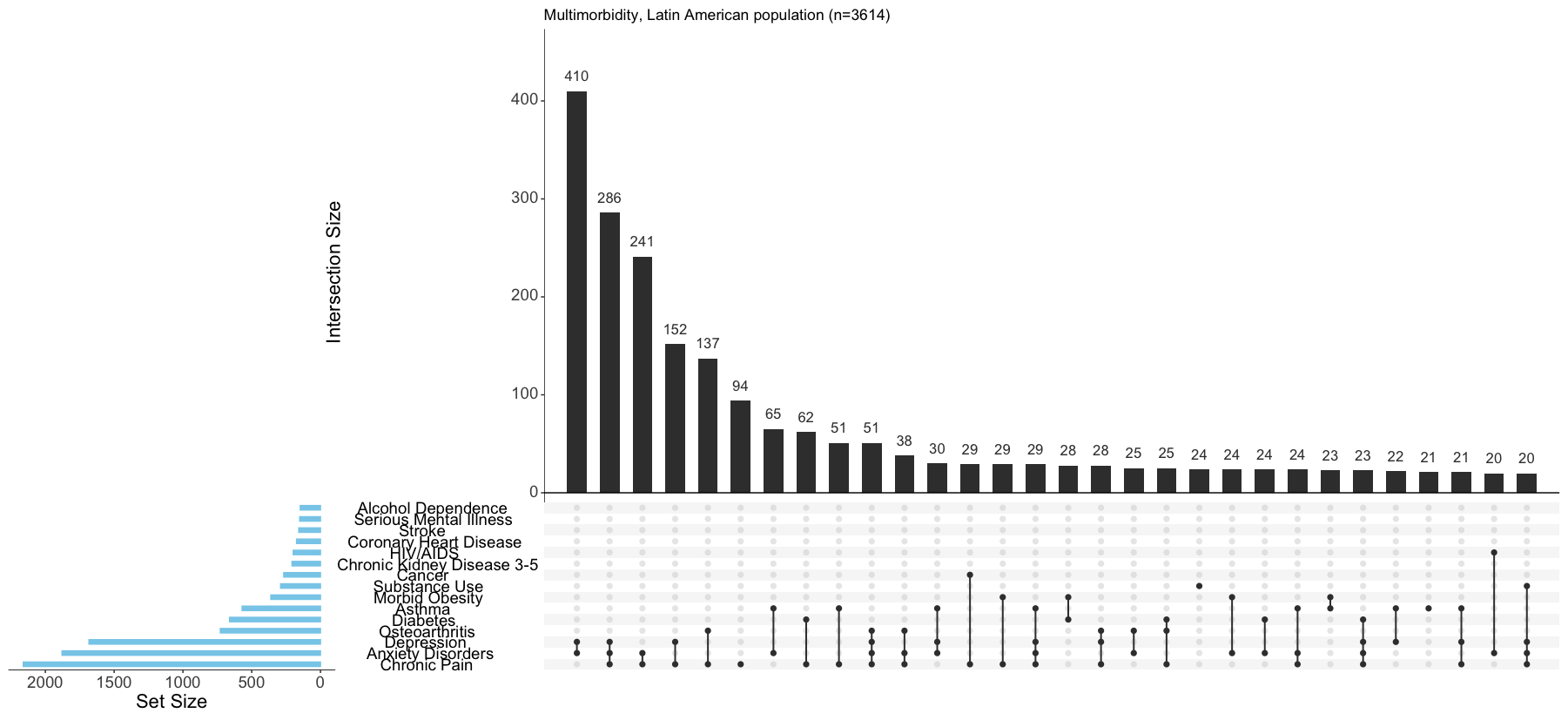


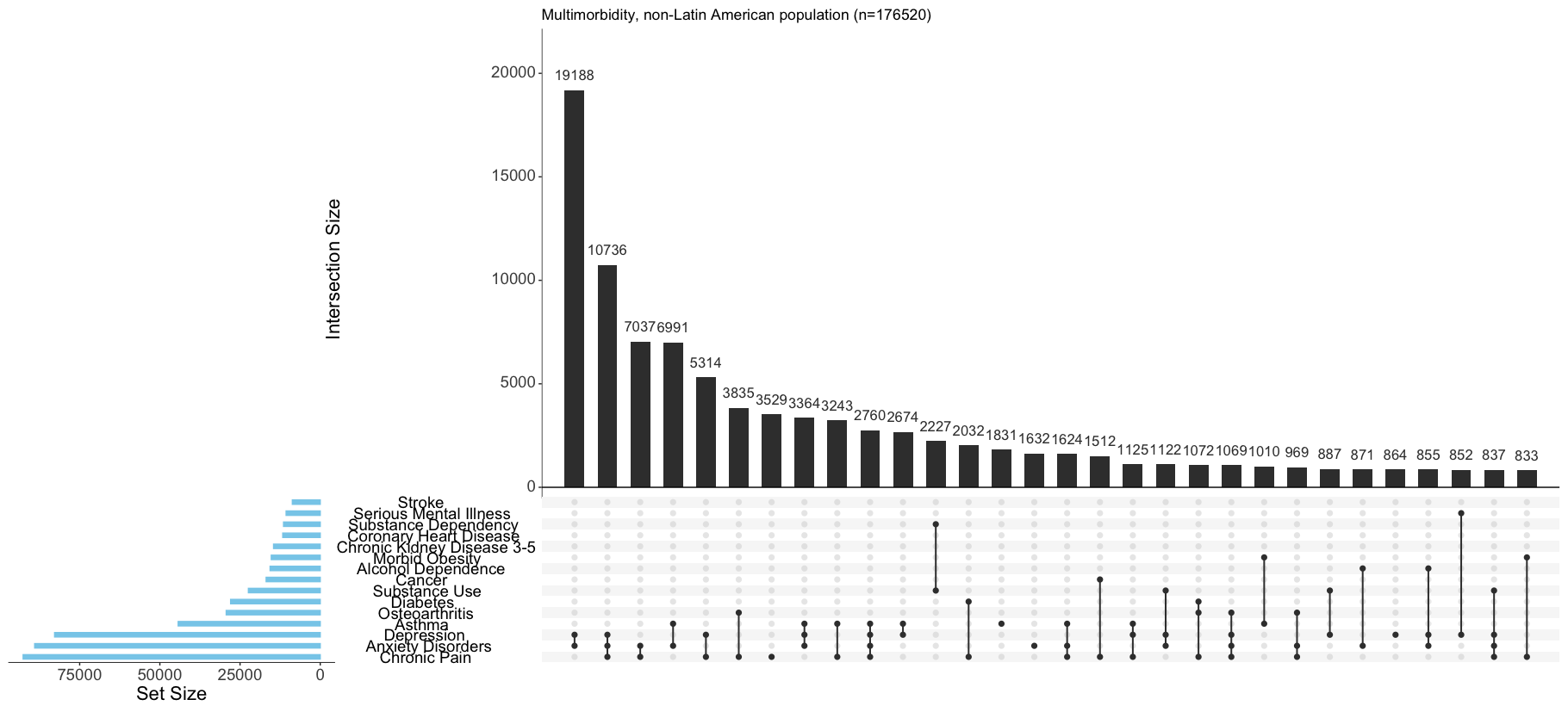


## Cumulative incidence of multimorbidity by ethnic group, 2005-2022. Multimorbidity was defined as having had two or more long-term conditions (LTCs) diagnosed on separate days during follow up. Counted LTCs are: alcohol dependence; atrial fibrillation; anxiety disorders; asthma; cancer; coronary heart disease; chronic kidney disease stages 3-5; COPD; chronic pain; dementia; depression; diabetes; epilepsy; heart failure; HIV/AIDS; inflammatory bowel disease; learning disabilities; liver disease; lupus; serious mental illness; morbid obesity; multiple sclerosis; osteoporosis; osteoarthritis; peripheral vascular disease; Parkinson's disease; rheumatoid arthritis; sickle-cell disease; stroke; substance dependency; transient ischemic attack; viral hepatitis B and C.

| Ethnic group | n | Cumulative incidence of multimorbidity (%) |
| --- | --- | --- |
| Black/African/Caribbean/Black British | 120067 | 26.7 |
| White | 287304 | 24.3 |
| Mixed/Multiple ethnic group | 31587 | 19.9 |
| Asian/Asian British | 56411 | 16.3 |
| Other ethnic group | 18654 | 14 |
| White Other | 185219 | 11.4 |
| Latin American | 28617 | 10.9 |
| Missing ethnic group data | 162579 | 9 |
