# Supplementary material for: Incidence of long-term conditions in the Latin American community of London: A validation and retrospective cohort study of 890,922 primary care records, 2005–2022
Source: PLoS One. 2024 Nov 27;19(11):e0312311. doi: 10.1371/journal.pone.0312311 (PMC11602084; doi:10.1371/journal.pone.0312311)
Supplement: S3 File — (DOCX) [file pone.0312311.s003.docx]

**S3 File.** Multivariable regression coefficients and sensitivity analyses for long-term conditions in the Latin American population.

## Table S3: Multivariable hazard ratios of 36 long-term conditions and risk factors. Each column is derived from a separate mixed effects competing risks regression model, with random effects terms for GP practice and lower super output area. Adjustment is made for sex, smoking and within-dataset deprivation quintile. N=579086.


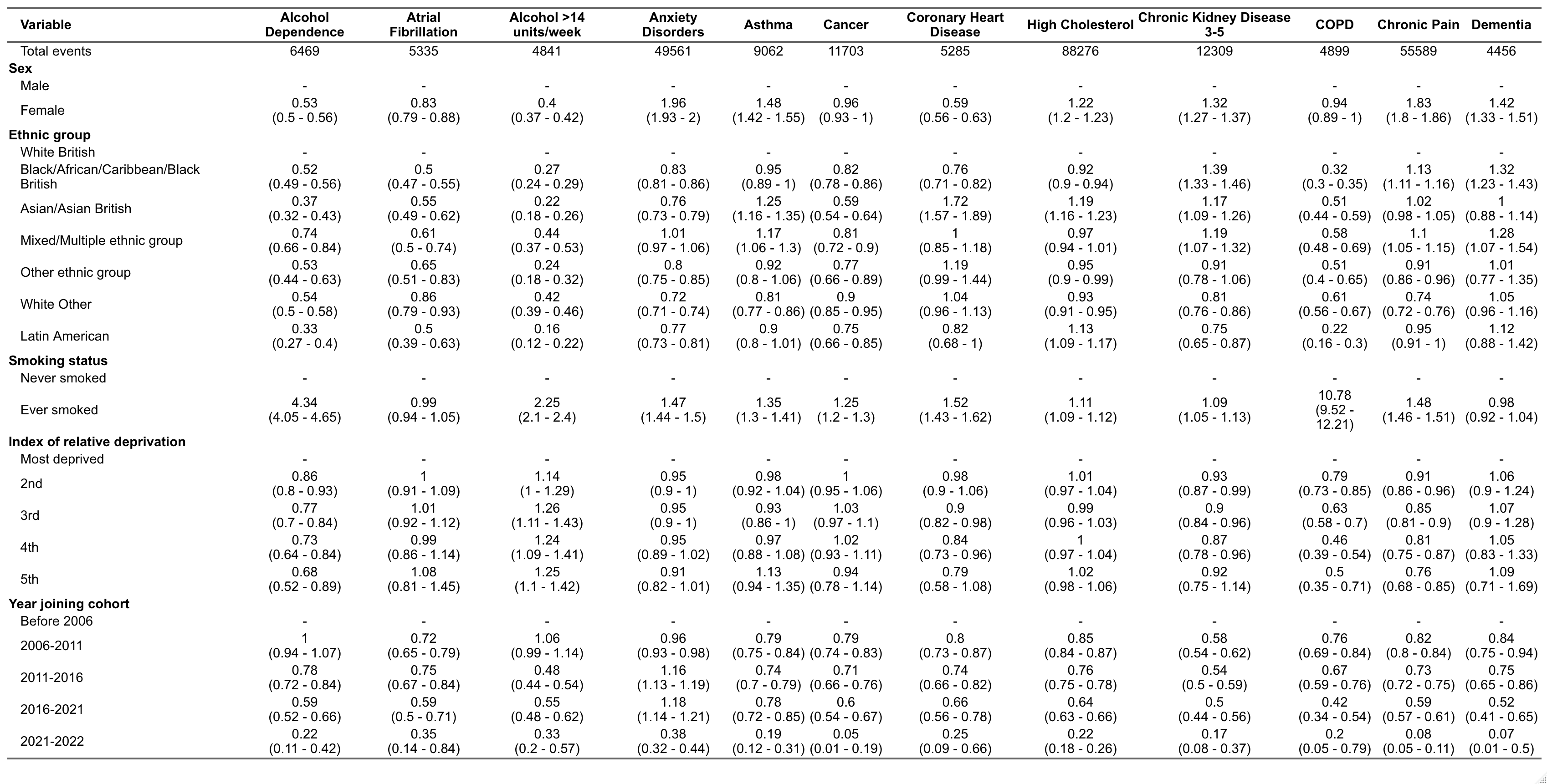


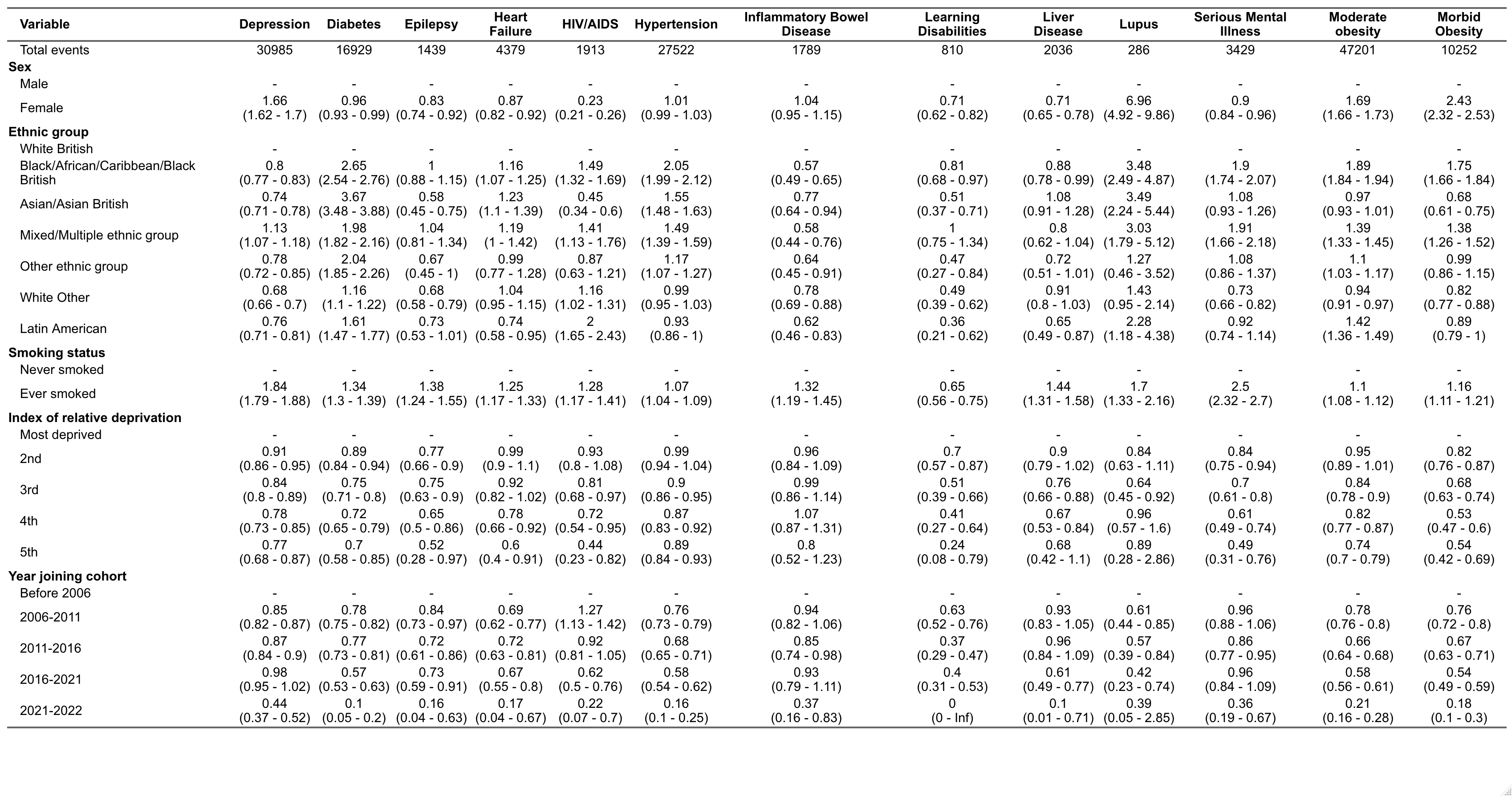


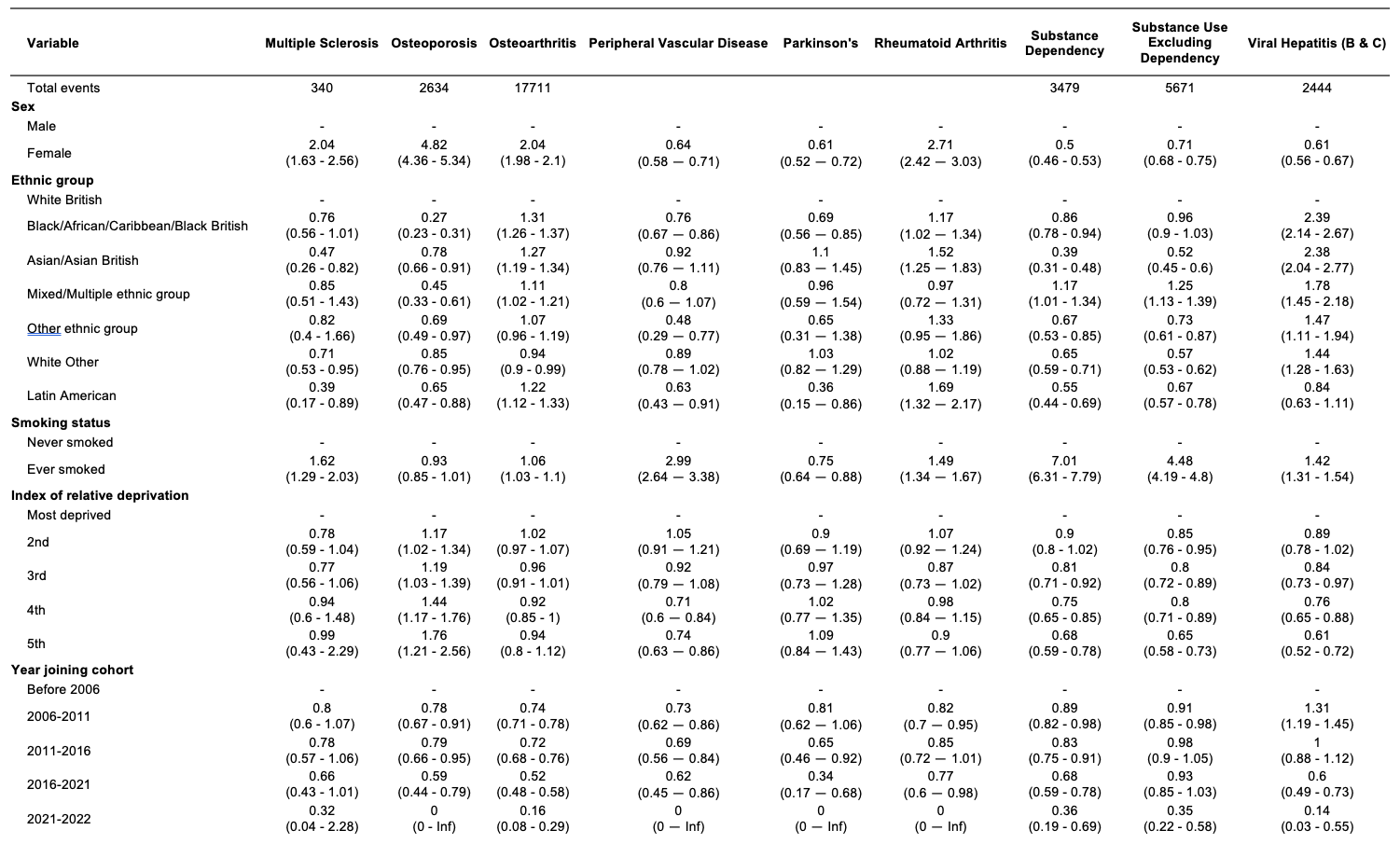


## Table S4: Sensitivity analysis of the effect of imputing missing ethnic group data as White British or Latin American. n=100,000, randomly sampled from the full population.

|  | **Multivariable hazard ratios for Latin American population** | | |
| --- | --- | --- | --- |
| **Long-term condition** | **Sample of 100,000**  **complete case analysis** | **Missing ethnicity data imputed as White British** | **Missing ethnicity data imputed as Latin American** |
| **Alcohol Dependence** | 0.31 (0.17 — 0.56) | 0.66 (0.54 — 0.81) | 0.45 (0.37 — 0.53) |
| **Atrial Fibrillation** | 0.75 (0.43 — 1.31) | 0.9 (0.7 — 1.15) | 0.47 (0.38 — 0.58) |
| **Anxiety Disorders** | 0.73 (0.63 — 0.85) | 0.86 (0.81 — 0.93) | 0.63 (0.59 — 0.67) |
| **Asthma** | 1.09 (0.79 — 1.5) | 1.07 (0.91 — 1.27) | 0.72 (0.62 — 0.84) |
| **Cancer** | 0.65 (0.44 — 0.95) | 1.11 (0.94 — 1.29) | 0.68 (0.6 — 0.79) |
| **Coronary Heart Disease** | 1.08 (0.65 — 1.8) | 1.29 (1.02 — 1.65) | 0.71 (0.58 — 0.89) |
| **Chronic Kidney Disease 3-5** | 1.1 (0.77 — 1.57) | 0.96 (0.79 — 1.15) | 0.55 (0.47 — 0.65) |
| **COPD** | 0.26 (0.11 — 0.63) | 0.7 (0.54 — 0.92) | 0.39 (0.31 — 0.49) |
| **Chronic Pain** | 1.1 (0.96 — 1.26) | 0.89 (0.82 — 0.96) | 0.57 (0.53 — 0.61) |
| **Dementia** | 1.3 (0.71 — 2.35) | 1.45 (1.1 — 1.91) | 0.65 (0.51 — 0.83) |
| **Depression** | 0.89 (0.74 — 1.06) | 0.8 (0.73 — 0.88) | 0.56 (0.52 — 0.61) |
| **Diabetes** | 1.63 (1.23 — 2.16) | 1.49 (1.28 — 1.75) | 0.84 (0.73 — 0.97) |
| **Epilepsy** | 0.6 (0.22 — 1.65) | 1.24 (0.83 — 1.85) | 0.63 (0.44 — 0.91) |
| **Heart Failure** | 0.91 (0.48 — 1.72) | 1.13 (0.85 — 1.5) | 0.68 (0.53 — 0.86) |
| **HIV/AIDS** | 3.1 (1.72 — 5.59) | 1.93 (1.33 — 2.8) | 1.28 (0.88 — 1.84) |
| **Inflammatory Bowel Disease** | 0.59 (0.21 — 1.62) | 0.83 (0.55 — 1.24) | 0.66 (0.46 — 0.94) |
| **Learning Disabilities** | 0.6 (0.18 — 1.96) | 0.58 (0.29 — 1.15) | 0.34 (0.2 — 0.58) |
| **Liver Disease** | 1.26 (0.65 — 2.46) | 1.3 (0.92 — 1.85) | 0.86 (0.63 — 1.19) |
| **Lupus** | 1.52 (0.18 — 12.51) | 1.65 (0.58 — 4.72) | 1.2 (0.43 — 3.34) |
| **Serious Mental Illness** | 1.21 (0.7 — 2.12) | 0.79 (0.58 — 1.09) | 0.61 (0.47 — 0.81) |
| **Morbid Obesity** | 0.81 (0.55 — 1.18) | 1 (0.84 — 1.21) | 0.68 (0.57 — 0.8) |
| **Multiple Sclerosis** | 0.76 (0.1 — 5.87) | 0.75 (0.29 — 1.9) | 0.79 (0.36 — 1.76) |
| **Osteoporosis** | 0.71 (0.33 — 1.54) | 1 (0.72 — 1.39) | 0.42 (0.31 — 0.56) |
| **Osteoarthritis** | 1.42 (1.12 — 1.8) | 1.18 (1.03 — 1.36) | 0.65 (0.57 — 0.74) |
| **Peripheral Vascular Disease** | 0.5 (0.16 — 1.6) | 1.07 (0.73 — 1.56) | 0.75 (0.55 — 1.03) |
| **Parkinson's** | 0 (0 — Inf) | 1.02 (0.5 — 2.1) | 0.62 (0.35 — 1.09) |
| **Rheumatoid Arthritis** | 1.87 (0.91 — 3.81) | 1.05 (0.66 — 1.66) | 0.79 (0.53 — 1.19) |
| **Stroke** | 1.08 (0.61 — 1.9) | 1.07 (0.81 — 1.42) | 0.68 (0.54 — 0.87) |
| **Substance Dependency** | 0.45 (0.22 — 0.91) | 0.56 (0.41 — 0.75) | 0.4 (0.31 — 0.52) |
| **Transient Ischemic Attack** | 1.22 (0.53 — 2.82) | 1.15 (0.76 — 1.74) | 0.73 (0.51 — 1.04) |
| **Viral Hepatitis (B & C)** | 0.75 (0.3 — 1.89) | 1.48 (1.04 — 2.11) | 1.24 (0.88 — 1.74) |
| **Hypertension** | 1.08 (0.87 — 1.35) | 1.13 (1 — 1.27) | 0.59 (0.53 — 0.65) |
| **Substance Use** | 0.7 (0.46 — 1.06) | 0.59 (0.48 — 0.72) | 0.44 (0.37 — 0.53) |
| **Alcohol >14 units/week** | 0.26 (0.14 — 0.51) | 0.55 (0.43 — 0.7) | 0.27 (0.21 — 0.34) |
| **High Cholesterol** | 1.15 (1.03 — 1.27) | 1.26 (1.19 — 1.34) | 0.58 (0.56 — 0.62) |
