# Supplementary material for: Incidence of long-term conditions in the Latin American community of London: A validation and retrospective cohort study of 890,922 primary care records, 2005–2022
Source: PLoS One. 2024 Nov 27;19(11):e0312311. doi: 10.1371/journal.pone.0312311 (PMC11602084; doi:10.1371/journal.pone.0312311)
Supplement: Attachment — Submitted filename: PLOS One rebuttal 08042024.docx [file pone.0312311.s006.docx]

Dear Dr Attia,

Thank you once again for your consideration of our manuscript ‘Incidence of long-term conditions in the Latin American community of London: a validation and retrospective cohort study of 890,922 primary care records, 2005-2022’. We are very grateful for the additional comments from peer reviewers who have very much helped to strengthen the manuscript. We have responded to each of the comments from peer reviewers in turn below.

I have also updated the filenames and author page according to the PLOS One guidance.

Thank you for noticing the licensing agreement for figure S4 in the text. I do apologise; the OpenStreetMap license we had quoted referred to its documentation rather than the map itself. The map is actually licensed for use under the Open Data Commons’ Open Database License, whose terms are compatible with the CCAL CC BY 4.0 licence. I have adapted the S1 File accordingly and uploaded a revised version of this file.

Yours sincerely on behalf of the co-authors,

Dr Jamie Scuffell

## Reviewer #1

Background Review:
The manuscript addresses an important gap in healthcare research by focusing on the health needs of minoritized populations, specifically the Latin American community in the United Kingdom. The background provides relevant context regarding the underrepresentation of Latin American individuals in health studies and highlights the diversity within this population. However, further discussion on the social determinants of health affecting Latin American communities in the UK could enrich the background section.

Many thanks for these comments. We have further contextualised the social determinants of health for the Latin American community by adding additional information on employment and educational status to lines 69-71 of the manuscript.

Lines 69-71 now read:

*Despite high levels of tertiary educational attainment, over a third of adults work in elementary jobs in London. They are more likely than other London residents to live in less secure private rented accommodation. [15]*

Lines 77-79 read:

*A mixed methods study of Latin American migrants in London demonstrated difficulties accessing primary care owing to language barriers and perceived discrimination from service providers. [19]*

Aim Review:
The aim of the study is clearly articulated, focusing on characterizing the incidence of long-term conditions (LTCs) and multiple LTCs (mLTCs) among the Latin American community in London. The research question is well-defined and addresses a significant public health issue, particularly in the context of superdiverse urban areas. However, providing more detail on the specific LTCs of interest and their relevance to the Latin American population would enhance the clarity of the aim.

Thank you. We have explained in the ‘outcomes’ section of the methods that the LTCs chosen were based on consensus exercises with local health stakeholders. We have clarified this further by describing the LTCs as ‘locally-important LTCs’ in line 82.

Line 81: *‘To do this we developed and validated a phenotype for Latin American ethnicity in routinely-collected data, comparing their life course incidence of locally-important long-term conditions (LTCs) to other ethnic groups.’*

Design and Setting Review:
The manuscript describes a retrospective cohort study utilizing pseudonymized primary care data from a large urban area of London spanning from 2005 to 2022. The choice of study design and setting is appropriate for capturing longitudinal health data and understanding healthcare utilization patterns among diverse populations. However, providing information on the primary care practices included in the study and any potential limitations associated with the use of electronic health records would strengthen the design and setting section.

Thank you for your thoughts on how to better describe the dataset. We had not made it clear that this is a sample of every adult registered to a primary care provider in a borough of London.

We have clarified the study sample in lines 86-89:

*Retrospective cohort study using Lambeth DataNet, a pseudonymised database derived from the electronic health records of every adult patient registered to a primary care provider in a superdiverse (45% non-White[13]) borough of London between 1^st^ April 2005 and 1^st^ February 2022. There are 40 primary care providers in the sample.*

Method Review:
The methods section outlines the identification of Latin American individuals using country of birth, language, and ethnicity codes, validated against national Census findings. The use of multivariable competing risks regression models to estimate the effect of ethnicity on LTC incidence is appropriate and statistically rigorous. However, providing details on how missing data or misclassification of LTCs were addressed in the analysis would enhance the transparency of the methodology.

Thank you. We explain in the methods that we conducted a sensitivity analysis for the exposure which was most likely to cause misclassification bias: missing ethnic group data. We have addressed the risk of misclassification of LTCs in an additional section in the discussion section of the paper.

Lines 259-264 now read:

*As this study used data from every care provider in a defined area, case ascertainment of many LTCs is likely to be high. HIV status in particular is well-recorded in this dataset owing to long-established opt-out testing in primary care and emergency departments. [33] Other conditions – risk factors, osteoarthritis, depression and anxiety – may be differentially measured between ethnic groups and therefore be prone to bias in either direction. Case ascertainment for some LTCs may be improved by a linkage to secondary care data.*

Results Review:
The results section presents key findings regarding LTC incidence among the Latin American population in London. The use of hazard ratios and confidence intervals effectively summarizes the associations between ethnicity and LTCs. However, providing absolute incidence rates or prevalence estimates for each LTC would enhance the interpretation of results and facilitate comparisons with other studies.

Thank you. We do describe cumulative incidence rates for each LTC in the Results section (lines 185–191). Given that the absolute incidence rates for LTCs will differ substantially by age, we have instead presented cumulative incidence function curves in Figures 3 and 4.

Conclusion Review:
The conclusion effectively summarizes the study's findings and emphasizes the importance of targeted health interventions for the Latin American community in London. The implications for public health practice and policy are clearly articulated, highlighting the potential for using primary care data to inform equitable health interventions. However, acknowledging potential limitations of the study, such as the reliance on primary care data and potential selection bias, would strengthen the conclusion's validity.

Thanks for these comments. As this is observational data we recognise the potential selection bias that can arise from using primary care data. However, as demonstrated in the study, a large proportion of Latin American residents of the borough of Lambeth – approximately 90% – are registered at a primary care practice. As this is the only means of accessing publicly-funded health services in the United Kingdom, there is likely good case ascertainment of these LTCs. We have further discussed this with additions to the limitations section., which now read:

Lines 259-264 now read:

*As this study used data from every care provider in a defined area, case ascertainment of many LTCs is likely to be high. HIV status in particular is well-recorded in this dataset owing to long-established opt-out testing in primary care and emergency departments. [33] Other conditions – risk factors, osteoarthritis, depression and anxiety – may be differentially measured between ethnic groups and therefore be prone to bias in either direction. Case ascertainment for some LTCs may be improved by a linkage to secondary care data.*

Overall, the manuscript contributes valuable insights into the health disparities experienced by the Latin American community in London and the need for tailored healthcare interventions. Strengthening the background with additional context, providing more detail on LTCs of interest, addressing potential limitations in the methodology, and acknowledging study limitations in the conclusion would enhance the manuscript's overall quality and impact.

Thank you for this feedback.

## Reviewer #2

*Reviewer 2 also offered specific feedback in comments on the manuscript and this has been very helpful to improve these areas of the manuscript. We have incorporated responses into this text and directly into the text of the manuscript.*

Thank you for the opportunity to review the authors’ work.

The authors introduce the subject of their study by providing an argument for the relevance of this work for other follow up studies.

Aim: The aim presented in the abstract differs in meaning to the aim stated at the end of the Introduction. One talks about describing the profile of long-term conditions in the Latin American community while the other also plans to describe health inequalities evidenced by the prevailing conditions in the Latin American community.

Thank you. Our aim was to describe some health inequalities faced by the Latin American community by considering differential incidence of long-term conditions by ethnicity. The LTCs we chose were chosen as being locally relevant. We have clarified this, as you have suggested, by focusing on incident conditions arising among the Latin American population (lines 79-81). Responding to the comment attached to lines 76-77, we have also clarified the aim in the Abstract so that it matches that in the introduction.

Lines 79-81 now read:

*We sought to better understand health inequalities affecting the Latin American population compared to other ethnic groups in an urban setting. To do this we developed and validated a phenotype for Latin American ethnicity in routinely-collected data, comparing their life course incidence of locally-important long-term conditions (LTCs) and multiple LTCs (mLTCs)* *to other ethnic groups.*

The aim in the Abstract now matches this:

*Characterise the incidence of long-term conditions (LTCs) and multiple LTCs (mLTCs) amongst the Latin American community of London. Compare the incidence of LTCs in the Latin American population to other ethnic groups.*

Methodology: A retrospective review of pseudonymized health data collated from GP practices is inadequate to effectively describe health inequalities as it does not collect enough information in this study to answer these questions.

Many thanks for your comments. We agree with your concerns of demonstrating causality from observational data such as primary care, which is prone to selection bias and residual confounding. However in multivariable analysis we were able to adjust for what are likely the most substantial confounders (age, sex, neighbourhood-level deprivation and between-practice variation in diagnosis). This is in keeping with the methodologies of other large-scale ethnic inequality studies, such as Mathur et al. (2021). We have added an explicit reference to this in the methods section (line 143):

*Each model was adjusted a priori for age, sex, smoking status (ever/never), and within-borough IMD quintiles, similarly to other large ethnic disparity studies using UK primary care data[3,4].*

We discuss the limitations of this in the discussion section, which we have further expanded in lines 259-264 as a result of your review.

Lines 259-264 now read:

*As this study used data from every care provider in a defined area, case ascertainment of many LTCs is likely to be high. HIV status in particular is well-recorded in this dataset owing to long-established opt-out testing in primary care and emergency departments. [33] Other conditions – risk factors, osteoarthritis, depression and anxiety – may be differentially measured between ethnic groups and therefore be prone to bias in either direction. Case ascertainment for some LTCs may be improved by a linkage to secondary care data.*

Studies based on UK primary care data have been shown to be representative of the overall UK population (Wolf et al. 2019). This study includes every patient registered to every practice in a borough of London and includes small-area neighbourhood deprivation indicators. We did not aim in this study to demonstrate causality. We have elaborated further on how UK deprivation indicators are calculated to make it clearer to international audiences how socioeconomic conditions are adjusted for in the analysis.

Lines 120-122 now read:

*The IMD is a weighted score of deprivation for an area with a resident population of between 1,000 and 3,000 covering the domains of education, housing, income, health, crime, housing and the living environment.*

Mathur R, Rentsch CT, Morton CE, Hulme WJ, Schultze A, MacKenna B, Eggo RM, Bhaskaran K, Wong AYS, Williamson EJ, Forbes H, Wing K, McDonald HI, Bates C, Bacon S, Walker AJ, Evans D, Inglesby P, Mehrkar A, Curtis HJ, DeVito NJ, Croker R, Drysdale H, Cockburn J, Parry J, Hester F, Harper S, Douglas IJ, Tomlinson L, Evans SJW, Grieve R, Harrison D, Rowan K, Khunti K, Chaturvedi N, Smeeth L, Goldacre B; OpenSAFELY Collaborative. Ethnic differences in SARS-CoV-2 infection and COVID-19-related hospitalisation, intensive care unit admission, and death in 17 million adults in England: an observational cohort study using the OpenSAFELY platform. Lancet. 2021 May 8;397(10286):1711-1724. doi: 10.1016/S0140-6736(21)00634-6. Epub 2021 Apr 30.

Achim Wolf, Daniel Dedman, Jennifer Campbell, Helen Booth, Darren Lunn, Jennifer Chapman, Puja Myles, Data resource profile: Clinical Practice Research Datalink (CPRD) Aurum, International Journal of Epidemiology, Volume 48, Issue 6, December 2019, Pages 1740–1740g, <https://doi.org/10.1093/ije/dyz034>

Results and Discussion: The authors provide a number of tables with adequate statistical analysis using multivariate regression analysis. The findings describe prevailing health conditions in the Latin American population in this community. The efforts of the authors to stretch the findings to describing health inequalities is inadequate as they do not have enough information and several confounding factors make it impossible to do so.

Many thanks. In this paper we did not want to estimate the causal effect of Latin American ethnicity on incidence of LTCs. Rather we wanted to provide pragmatic data and a reproducible methodology for policy-makers to identify ethnic disparities using primary care data. We have clarified this further in the Discussion (lines 235-236) which now read: ‘We provide a reproducible methodology to describe health inequalities in LTC diagnoses for minoritised populations.’

In the study we provide both data on prevailing conditions for the Latin American population, as well as multivariable relative incidence rates with respect to ethnicity. As a result of your comments we have changed the wording to make it more explicit that this is a descriptive study and not a causal one (line 233, 307-310).

Line 233: ‘This study describes the incidence of common LTCs in the Latin American population. – a minoritised group who identify across several census ethnic groups.’

Lines 307-310:

*This work provides a reproducible methodology to describe chronic health disparities in minoritised urban populations over time, after adjustment for common confounding factors. Here we describe especially high rates of HIV/AIDS amongst the Latin American community in London, as well as increased rates of rheumatological disorders and diabetes.*

Strength and Limitations: The authors have described in some detail the many limitations to this study. The very reasons the conclusion is not adequate is described in this section, as they highlight the challenges of missing data, confounders and not enough information collated based on the study method.

In this revision we have further expanded the strengths and limitations section to encompass your comments

Lines 259-264 now read:

*As this study used data from every care provider in a defined area, case ascertainment of many LTCs is likely to be high. HIV status in particular is well-recorded in this dataset owing to long-established opt-out testing in primary care and emergency departments. [33] Other conditions – risk factors, osteoarthritis, depression and anxiety – may be differentially measured between ethnic groups and therefore be prone to bias in either direction. Case ascertainment for some LTCs may be improved by a linkage to secondary care data.*

The conclusion: I recommend the authors conclude on the point of describing the profile of illnesses in the different ethnic groups identified in the study, the study is not powered enough to conclude on the health inequalities experienced by the Latin American population in comparison to other ethnic groups in the study population.

Thank you for your thoughts. This study includes the entire primary care registered population of a particular UK borough. Given that primary care registration is very high in the UK it is likely the data are representative of the population studied and this has been shown in previous studies e.g. Wolf et al. (2019)

Although we did not conduct formal power calculations as the dataset is so large and there are varying prevalences of LTCs, the confidence intervals demonstrate clearly that we were able to estimate effect sizes with appropriate precision to ensure that the results are meaningful.

We make reference to the inherent complexity of ethnic determinants of health in the discussion section, where unmeasured structural factors are likely at play.

We believe that, notwithstanding important unmeasured confounders and mediators with you have identified, the paper adds to the health inequality and migrant health literature in urban populations. As a result of your comments we have either defined the word ‘health inequalities’ or used the term ‘health disparities’ to remove any misunderstanding that this study aimed to answer causal health inequality questions. The Conclusion (line 307) now reads *‘This work provides a reproducible methodology to describe chronic health disparities in minoritised urban populations over time, after adjustment for common confounding factors.’*

Achim Wolf, Daniel Dedman, Jennifer Campbell, Helen Booth, Darren Lunn, Jennifer Chapman, Puja Myles, Data resource profile: Clinical Practice Research Datalink (CPRD) Aurum, International Journal of Epidemiology, Volume 48, Issue 6, December 2019, Pages 1740–1740g, <https://doi.org/10.1093/ije/dyz034>

Reference: This is done correctly in the Vancouver style as required by the journal. The literature presented is recent and relevant to the population where the study was done.

Thank you.

Please see manuscript for comments on the sections.

Thank you especially for your comments on the discussion section which is copied below and responded to in turn:

*Line 233:* This statement suggests a direct cause and effect relationship. Research already describes several environmental factors that influence outcomes and a lot of these are not necessarily due to the social determinants of health as the authors suggest.

Thank you for this detailed feedback. Here we aimed to describe associations and not a causal relationship, and we have edited the sentence to remove suggestions that smoking or alcohol was part of the mechanism that might influence obesity, diabetes and hypercholesterolaemia incidence.

The findings still are not clearly describing what the authors were hoping to show. The aim is unclear. The aim needs to inform of what the authors question was, the findings and other steps can then be appraised to see if the researchers' question has been answered.

Thank you for the comments about the aims. We have revised the wording for this to increase the clarity of what we were trying to achieve with the research question (Aim in abstract, last paragraph of introduction section).

Lines 79-81 now read:

*We sought to better understand health inequalities affecting the Latin American population compared to other ethnic groups in an urban setting. To do this we developed and validated a phenotype for Latin American ethnicity in routinely-collected data, comparing their life course incidence of locally-important long-term conditions (LTCs) and multiple LTCs (mLTCs)* *to other ethnic groups.*

The aim in the Abstract now matches this:

*Characterise the incidence of long-term conditions (LTCs) and multiple LTCs (mLTCs) amongst the Latin American community of London. Compare the incidence of LTCs in the Latin American population to other ethnic groups.*

The authors have made descriptions highlighting increased risk among the Latin American popln without showing that the prevalence of these health conditions is enough to state the popln is at increased risk due to health inequalities. The prevalence of HIV in this study popln does not immediately interpret to health inequality as other environmental factors could explain the prevalence of HIV in the study population.

Thank you for your comments. We have described the life-course cumulative incidence of each long-term condition in the Latin American population in figure 3 (compared to non-Latin Americans) and figure 4 (over time, compared to both White and Black ethnic group). These absolute figures should contextualise the clinical significance of increased health risks. The study was adequately powered to detect increased risks of health conditions in this group compared to the White and Black ethnic groups.

We agree that there is not necessarily a causal association between being Latin American and having higher HIV incidence. We have acknowledged that there are unmeasured confounders, particularly differences in proportions of gay and bisexual men who are the main group of people who live with HIV in London. (lines 232-236).

*Line 239:* This statement gives an impression that the authors are trying to make a finding that does not fit into the narrative suggested in the conclusion into the mold as with other findings. Again the lower rates may be due to under reporting as they say but mental health conditions according to research is under reported in all populations in varied settings. Why do the authors consider under reporting for only the Latin American popln in this study and not the others?

Thank you. We have expanded the discussion on undermeasurement by ethnic group in lines 259-264, including a comment on differential measurement of mental health conditions. The comparison we wished to make in this line related to relative rather than absolute comparisons and we have adjusted the sentence to aim to make this clearer.

Lines 259-264, as described above, now read:

*The dataset is inclusive and representative of the urban population studied. As this study used data from every care provider in a defined area, case ascertainment of many LTCs is likely to be high. HIV status in particular is well-recorded in this dataset owing to long-established opt-out testing in primary care and emergency departments. [33] Other conditions – risk factors, osteoarthritis, depression and anxiety – may be differentially measured between ethnic groups and therefore be prone to bias in either direction. Case ascertainment for some LTCs may be improved by a linkage to secondary care data.*

*Line 262:* This statement in line 255-257 can be adequate explanation for all the differences observed between ethnic populations in this study. The findings are not sufficient to suggest health inequalities.

Thank you. In the multivariable analysis we adjusted for the most common sociodemographic non-modifiable confounders likely to confound the effect of being Latin American on the incidence of each long-term condition. We agree that unmeasured confounding is a limitation of the study and we have addressed this in more detail in lines 261-267.

*Line 275:* This statement by the authors also gives strong contradiction to any statement giving health inequalitities [sic] as an explanation for the increased rates of certain LTC compared to others.

We agree that, as with all studies of ethnic determinants of health, the mediators of the relationship act at multiple levels which are rarely measurable reliably, accurately and simultaneously. In this case we adjusted for the most common patient-level determinants of health (age, sex, smoking status) and neighbourhood-level determinants of health (deprivation and GP practice). This is in keeping with many other large scale studies of ethnic determinants of health, for example, for COVID-19 (Mathur et al. 2021), diabetes (Mathur et al. 2020) and hypertension (Basta et al. 2024). We have acknowledged these limitations in the discussion section.

We also recognise that, for an international audience, it would be helpful to explain and clarify how deprivation was measured in this study. We have therefore added a sentence to explain this. Line 120-122 now reads:

*The IMD is a weighted score of deprivation for an area with a resident population of between 1,000 and 3,000 covering the domains of education, housing, income, health, crime, housing and the living environment.*

Basta, Karol; Ledwaba-Chapman, Lesedi; Dodhia, Hiten; Ashworth, Mark; Whitney, David; Dalrymple, Kathryn; Wang, Yanzhong. Hypertension prevalence, coding and control in an urban primary care setting in the UK between 2014 and 2021. Journal of Hypertension 42(2):p 350-359, February 2024. | DOI: 10.1097/HJH.0000000000003584

Mathur R, Rentsch CT, Morton CE, Hulme WJ, Schultze A, MacKenna B, Eggo RM, Bhaskaran K, Wong AYS, Williamson EJ, Forbes H, Wing K, McDonald HI, Bates C, Bacon S, Walker AJ, Evans D, Inglesby P, Mehrkar A, Curtis HJ, DeVito NJ, Croker R, Drysdale H, Cockburn J, Parry J, Hester F, Harper S, Douglas IJ, Tomlinson L, Evans SJW, Grieve R, Harrison D, Rowan K, Khunti K, Chaturvedi N, Smeeth L, Goldacre B; OpenSAFELY Collaborative. Ethnic differences in SARS-CoV-2 infection and COVID-19-related hospitalisation, intensive care unit admission, and death in 17 million adults in England: an observational cohort study using the OpenSAFELY platform. Lancet. 2021 May 8;397(10286):1711-1724. doi: 10.1016/S0140-6736(21)00634-6. Epub 2021 Apr 30.

Mathur R, Palla L, Farmer RE, Chaturvedi N, Smeeth L. Ethnic differences in the severity and clinical management of type 2 diabetes at time of diagnosis: A cohort study in the UK Clinical Practice Research Datalink. Diabetes Res Clin Pract. 2020 Feb;160:108006. doi: 10.1016/j.diabres.2020.108006. Epub 2020 Jan 7. PMID: 31923438; PMCID: PMC7042884.

*Line 286:* The authors have not provided enough information to come to this conclusion. The work should be reconsidered as a description of the profile of diseases noted to be prevalent in the varied ethnic populations in the area studied.

Thank you for this comment which is also alluded to in the full text. We have changed line 301 in the conclusion to make it clearer that this is a descriptive paper. It now reads: ‘This work provides a reproducible methodology to describe chronic health disparities in minoritised urban populations over time, after adjustment for common confounding factors.’

Thank you.

Reviewer.
