# Supplementary material for: Incidence of long-term conditions in the Latin American community of London: A validation and retrospective cohort study of 890,922 primary care records, 2005–2022
Source: PLoS One. 2024 Nov 27;19(11):e0312311. doi: 10.1371/journal.pone.0312311 (PMC11602084; doi:10.1371/journal.pone.0312311)
Supplement: Attachment — Submitted filename: Reviewer comments PONE-D-24-04965.docx [file pone.0312311.s005.docx]

Reviewer comments PONE-D- 24-04965

Thank you for the opportunity to review the authors’ work.

The authors introduce the subject of their study by providing an argument for the relevance of this work for other follow up studies.

Aim: The aim presented in the abstract differs in meaning to the aim stated at the end of the Introduction. One talks about describing the profile of long-term conditions in the Latin American community while the other also plans to describe health inequalities evidenced by the prevailing conditions in the Latin American community.

Methodology: A retrospective review of pseudonymized health data collated from GP practices is inadequate to effectively describe health inequalities as it does not collect enough information in this study to answer these questions.

Results and Discussion: The authors provide a number of tables with adequate statistical analysis using multivariate regression analysis. The findings describe prevailing health conditions in the Latin American population in this community. The efforts of the authors to stretch the findings to describing health inequalities is inadequate as they do not have enough information and several confounding factors make it impossible to do so.

Strength and Limitations: The authors have described in some detail the many limitations to this study. The very reasons the conclusion is not adequate is described in this section, as they highlight the challenges of missing data, confounders and not enough information collated based on the study method.

The conclusion: I recommend the authors conclude on the point of describing the profile of illnesses in the different ethnic groups identified in the study, the study is not powered enough to conclude on the health inequalities experienced by the Latin American population in comparison to other ethnic groups in the study population.

Reference: This is done correctly in the Vancouver style as required by the journal. The literature presented is recent and relevant to the population where the study was done.

Please see manuscript for comments on the sections.

Thank you.

Reviewer.
